# PULMONARY CANDIDIASIS PRESENTING AS MYCETOMA

**DOI:** 10.4103/0970-2113.45285

**Published:** 2008

**Authors:** Arshad Aitaf Bachh, Inaamul Haq, Rahul Gupta, HG Varudkar, Mohan B Ram

**Affiliations:** 1 Assistant Professor, Department of Pulmonary Medicine, Mamata Medical College and Hospital, Khammam, Andhra Pradesh, India; 2 Resident, Department of Pulmonary Medicine, Mamata Medical College and Hospital, Khammam, Andhra Pradesh, India; 3 Professor, Department of Pulmonary Medicine, Mamata Medical College and Hospital, Khammam, Andhra Pradesh, India

**Keywords:** Mycetoma, Candida, Haemoptysis

## Abstract

Candida is a saprophytic yeast that is frequently recovered from the respiratory tract. Most mycetoma lesions are due to Aspergillus species growing inside an existing cavity. The saprophytic nature of the Candida species in the human respiratory tract obscures diagnosis of Candida pulmonary infections. Only a few cases of mycetoma due to Can-dida have been reported. We report a case of mycetoma caused by Candida albicans in a diabetic immunocompromised tuberculous patient. Diagnosis was confirmed by biopsy and certain points strongly favoured the diagnosis. The patient was immunocompromised due to uncontrolled diabetes mellitus. Candida albicans was grown from bronchial and repeatedly from sputum samples and Candida antigen was positive from blood in high titre. There was a good clinical as well as radiological response to antifungal therapy and Candida antigen became negative. We emphasize that in the immunosuppressed host, a mycetoma-like lesion may be caused by Candida pulmonary infection.

## INTRODUCTION

A mycetoma is a conglomeration of intervened fungal hyphae admixed with mucous and cellular debris within a pulmonary cavity or ectatic bronchus. Historically, the most common underlying cause has been tuberculosis and Aspergillus species, the most common colonizing fungus.^1^ Pulmonary disease caused by Candida species is rare and its saprophytic nature in the human respiratory tract obscures diagnosis as well. Therefore, the definitive diagnosis of pulmonary candidiasis is based on demonstration of the fungus in lung tissue with associated inflammation. Only a few cases of mycetoma due to Candida have been reported. We report a case of mycetoma caused by Candida albicans in a diabetic immunocompromised tuberculous patient.

## CASE REPORT

A 50 year-old male diabetic patient, a smoker and a chronic alcoholic, was admitted in March 2007 with fever, cough, left pleuritic chest pain, frank haemoptysis, loss of appetite, general weakness and uncontrolled diabetes. The duration of current symptoms was four weeks. He had completed nine months of antitubercular therapy three years back as he was diagnosed a case of sputum smear positive pulmonary tuberculosis in 2003.

The patient was clinically febrile (38.5°C) with left upper lobe consolidation. Laboratory examination showed normal white blood cells with leucocytosis. Total leucocyte count was 12,500/cumm with neutrophils 78%, lymphocytes 18% and eosinophils 4%. Fasting blood glucose was 230 mg/dL. Renal and liver function tests were within normal limits. X-ray chest PA view was suggestive of non-homogenous opacity in left upper zone (Fig. I.

**Fig. 1:**
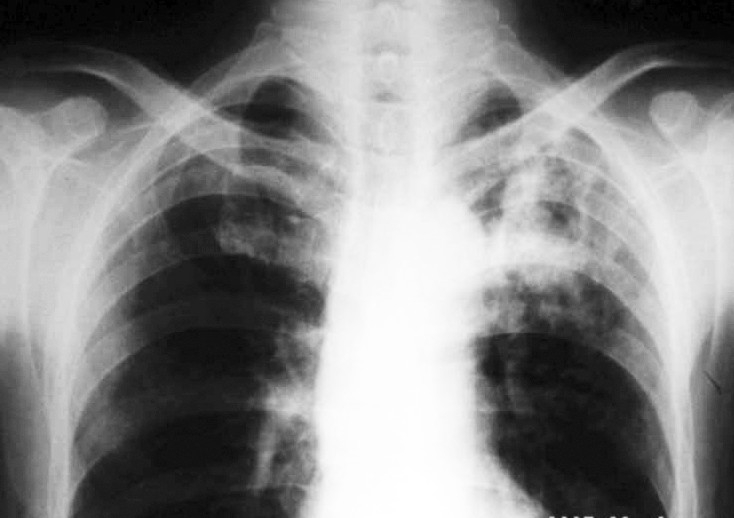
X-ray chest PA view showing non-homogenous opacity, left upper zone

Sputum smear for acid-fast bacilli (AFB) was negative. Culture of sputum for AFB was negative. Blood culture was also negative. He was using insulin for his diabetes. The patient was put on MSI (Multiple Subcutaneous Insulin) regimen with human insulin (regular) 8 IU each, half hour before breakfast, lunch & dinner and 12 IU of NPH insulin subcutaneously at bedtime. Trial of antibiotics with ceftriaxone 1 gm IV 12 hourly and clindamycin 300 mg IV six hourly was given with no improvement. Bronchoscopy was performed which showed external compression of left upper lobe bronchus, with inflamed mucosa. Bronchial specimens were negative for malignant cells, AFB and for pyogenic organisms but showed moderate growth of Candida albicans. Sputum examination for fungus also showed profuse growth of Candida albicans in consecutive samples. Two blood samples for Candida antigen were positive with titre value of 1:16. Computed Tomography (CT) scan of chest with contrast showed fibrotic lesions in both lungs. There was a cavity lesion with solid component in its dependent portion giving rise to crescent sign in apicoposterior segment of left upper lobe (Fig. II). Left upper lobe bronchus was compressed by peribronchial thickening. CT guided transthoracic needle aspiration was done. The culture of the aspirated sample yielded a moderate amount of Candida albicans. Culture for aerobic, anaerobic and mycobacteria was negative. Transthoracic tissue biopsy showed yeasts with pseudo-hyphae in a necrotic area with granulocyte infiltration. The patient was put on injection Amphotericin-B (30 mg IV per day) for three weeks. After that he improved clinically as well as radiologically (Fig. III & IV). He was then discharged from hospital on oral ketoconazole (400 mg per day). A repeat blood test for Candida antigen was negative in two consecutive samples after completing the course of antifungal treatment for a total of 60 days. He remained well at eight months follow-up.

**Fig 2:**
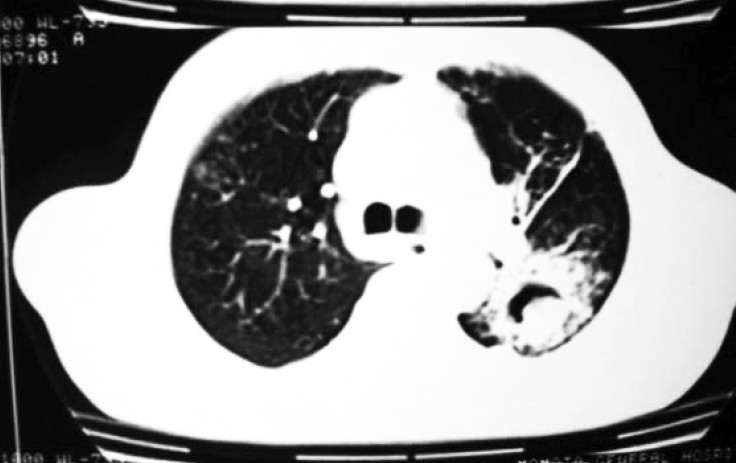
CT thorax showing mycetoma with air crescent in left apico-posterior segment.

**Fig 3:**
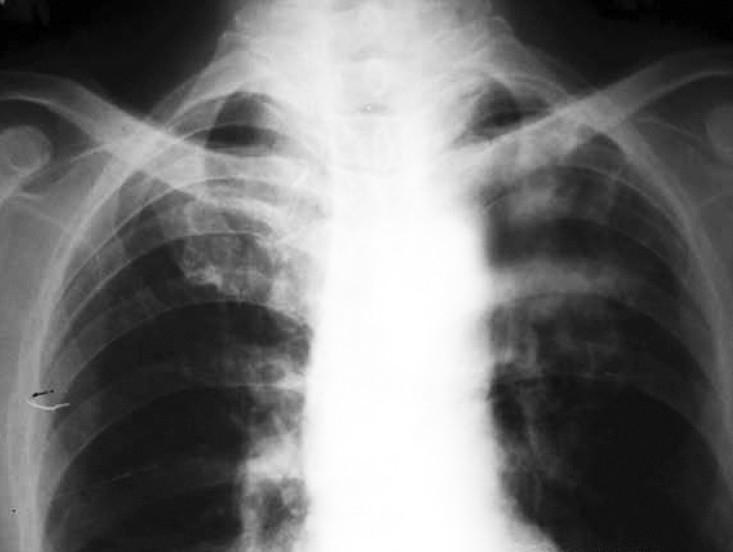
X-ray chest PA view post-treatment showing improvement in non-homogenous opacity, left upper zone.

**Fig 4:**
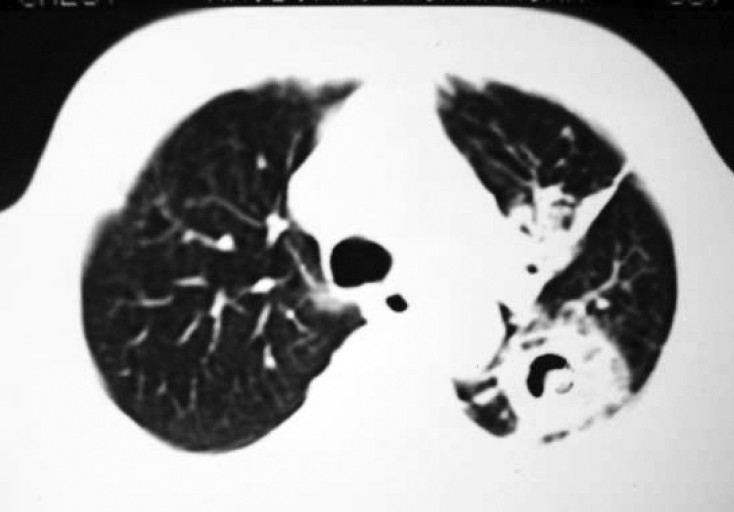
Post-treatment CT thorax showing decreased size of mycetoma with air crescent and increased fi brotic changes.

## DISCUSSION

Pulmonary candidiasis is a rare condition that usually occurs in immunosuppressed patients.^2^ Presence of Candida in respiratory specimens may be due to contamination and there is no specific clinical or radiological picture. Conclusive diagnosis requires demonstration of the organism in tissues.^3^ Pulmonary invasion results either due to dissemination or aspiration from upper airways. The second possibility is more likely in our case as this mode of infection is particularly common in chronic debilitating disease.^3^,^4^

This case was confirmed by biopsy and certain points strongly favour our diagnosis. The patient was immunocompromised due to uncontrolled diabetes mellitus. Candida albicans was grown from bronchial and repeatedly from sputum samples and Candida antigen was positive from blood in high titre. Sputum and bronchial specimen cultures were negative for pyogenic organisms as well as AFB. Blood culture for pyogenic organisms was negative. The patient failed to respond to ordinary antibiotics. CT scan showed a cavity with fungus ball in it. Candida antigen became negative and there was a good clinical as well as radiological response to antifungal therapy. Candida antigen detection was done using a latex agglutination kit. The kit is based on a heat-labile cytoplasmic antigen and is specific in the presence of high titres (1≥4 is significant).^5^

On chest X-ray, pulmonary candidiasis has been observed as bilateral bronchopulmonary infiltrates, a large cavitation and even pulmonary abscess.^2^,^6^ Pulmonary candidiasis can be present in four clinicopathologic forms: (a) the disease can exist as a part of systemic haematogenous infection associated with multiorgan involvement and a primary extrapulmonary site; (b) pulmonary candidiasis can occur as a primary infection that is presumably acquired by aspiration of organisms from the oral cavity. The infection may remain limited to the lung or may eventually disseminate; (c) the organism can accumulate in the airways in association with pulmonary disease; and (d) it can occasionally manifest by a mycetoma.^4^ In our case Candida presented as mycetoma in a pre-existing tuberculous cavity. Mycetoma due to Candida is a rare condition. Only few cases have been reported (Table I).^2^,^3^,^7^,^8^,^9^ We emphasize that in the immunosuppressed host, a mycetoma-like lesion may be caused by Candida pulmonary infection.

**Table 1:** Details of case reports of Candida species as a cause of pulmonary mycetoma.

S. No.	Author(s) Year	Age (years) Sex	Immune Status (Nature)	Method(s) of Diagnosis	Treatment Modality	Follow Up
1	Watanakunakorn C^8^. (1983)	33 Male	Imunocompromised (AML)	TTNA under fluoroscopy Culture	Amphotericin B IV Flucytosine Ketoconazole	Resolution on CXR after 10weeks
2	Prats E, Sans J, Valldeperas J, et al^2^ (1995)	62 Male	Immunocompromised (Post RT for T4N2M1 Squamous cell CA right superior sulcus)	Postmortem TTNA & biopsy culture	-	-
3	Shelly MA, Poe RH, Kapner LB^9^ (1996)	50 Male	Immunocompetent	Bronchoscopy under fluoroscopic guidance brush, BAL & biopsy culture	No specific treatment	CXR unchanged at 8 months At 24 months patient asymptomatic
4	Abel AT, Parwer S, Sanyal SC3 (1998)	58 Male	Immunocompromised (Diabetes mellitus, Drug resistant tuberculosis)	Bronchoscopic specimen culture Blood candida antigen positive	Amphotericin B IV ketoconazole	CT & CXR at 60 days complete resolution At 8 month patient

AML=Acute myeloid leukemia, TTNA=Transthoracic needle aspiration, CXR=Chest X-ray, RT=Radiotherapy, CA=Carcinoma, BAL=Bronchoalveolar lavage, CT=Computed tomography.
